# Supplementary figures and images for: Protein prediction for trait mapping in diverse populations
Source: PLoS One. 2022 Feb 24;17(2):e0264341. doi: 10.1371/journal.pone.0264341 (PMC8870552; doi:10.1371/journal.pone.0264341)

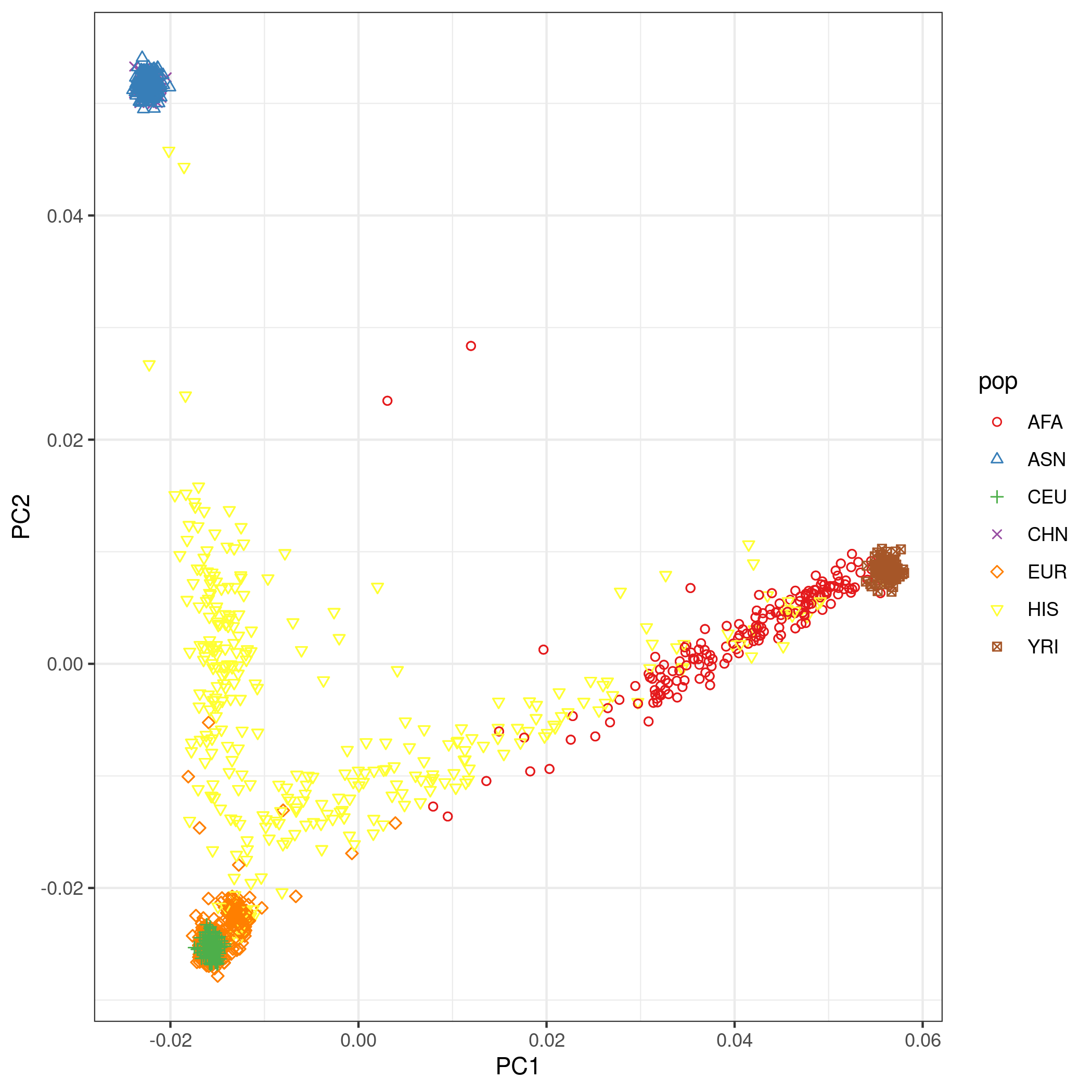

Supplement: S1 Fig — Biplot of the first two principal components of TOPMed MESA populations with 1000 Genomes reference populations. Genetic PCs of TOPMed participants with both genomic and proteomic data were estimated with PCAIR. Pop codes: TOPMed African American (AFA), TOPMed Chinese (CHN), TOPMed European (EUR), TOPMed Hispanic (HIS), 1000 Genomes East Asians from Beijing, China and Tokyo, Japan (ASN), 1000G European ancestry from Utah (CEU), and 1000G Yoruba from Ibadan, Nigeria (YRI). (TIF) [file pone.0264341.s001.tif]

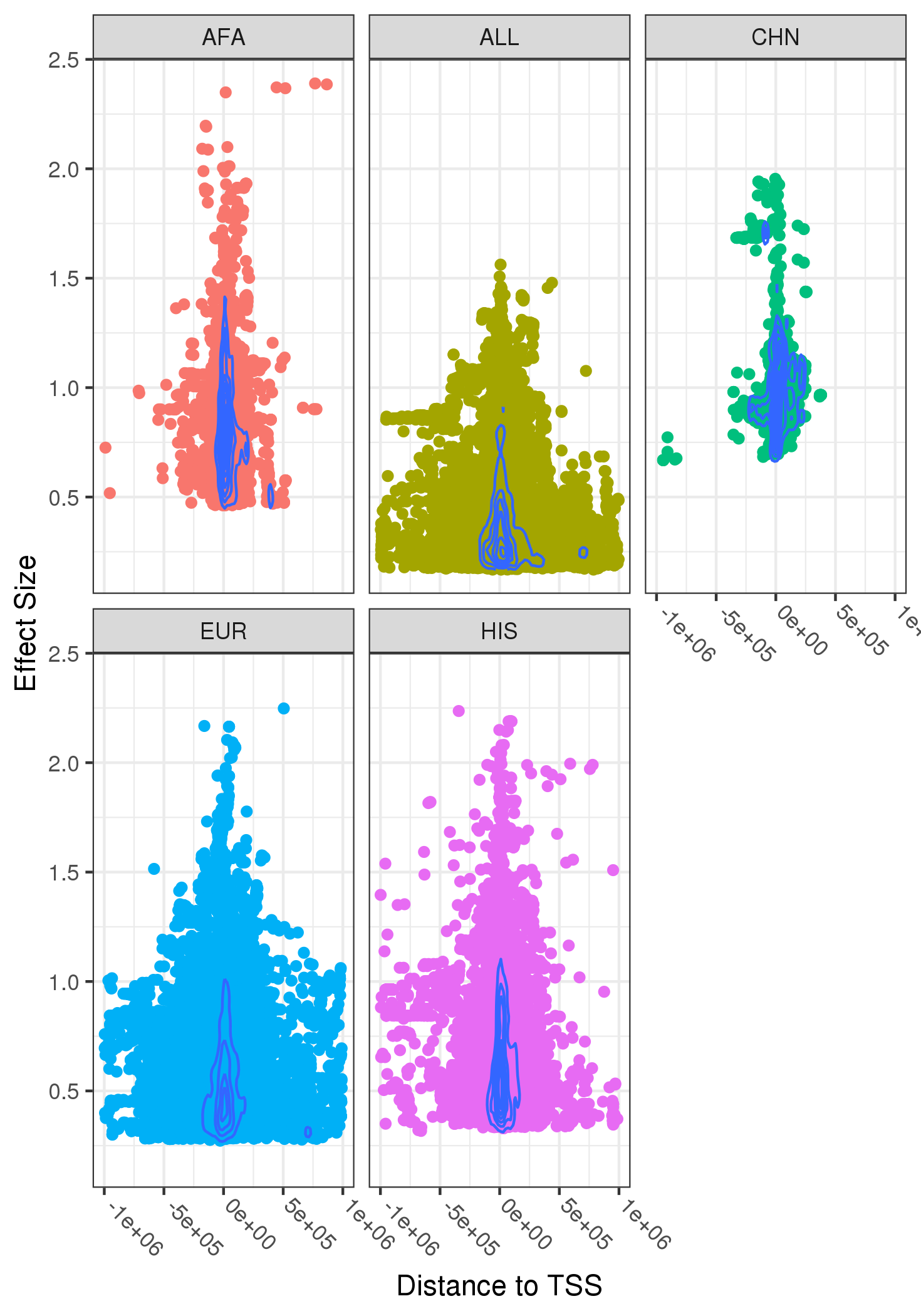

Supplement: S2 Fig — Significant pQTL (FDR <0.05) effect sizes are plotted versus the SNP distance to the TSS of the protein encoding gene in each TOPMed MESA population. Contour lines from two-dimensional kernel density estimation show pSNPs are concentrated at the TSS in all populations. (TIF) [file pone.0264341.s002.tif]

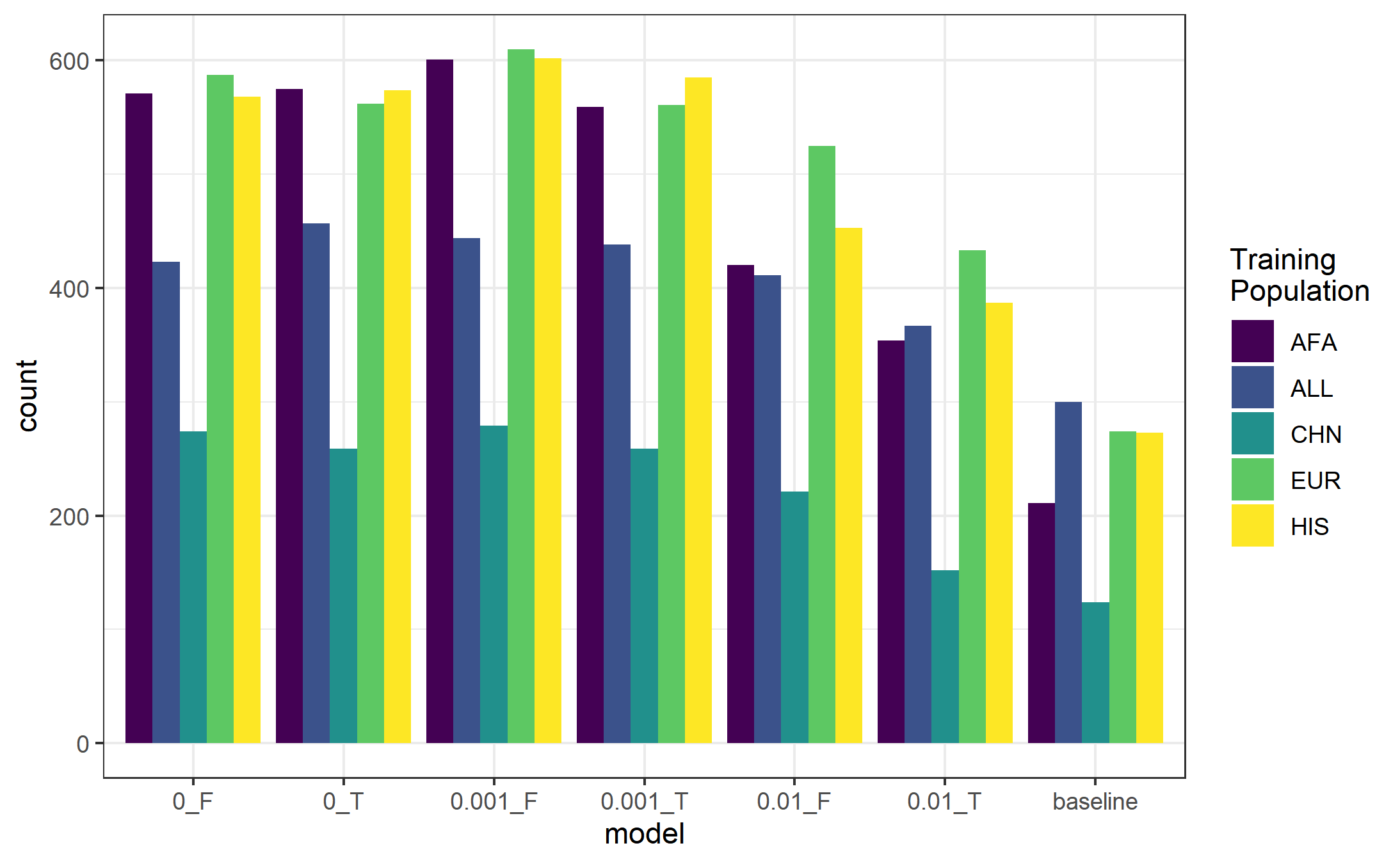

Supplement: S3 Fig — In total 1238 unique protein aptamers have significant prediction models (ρ > 0.1, p < 0.05) across all strategies and training populations. Number of significant protein models scales approximately with sample size of the training population, with the exception of ALL fine-mapped models. (TIF) [file pone.0264341.s003.tif]

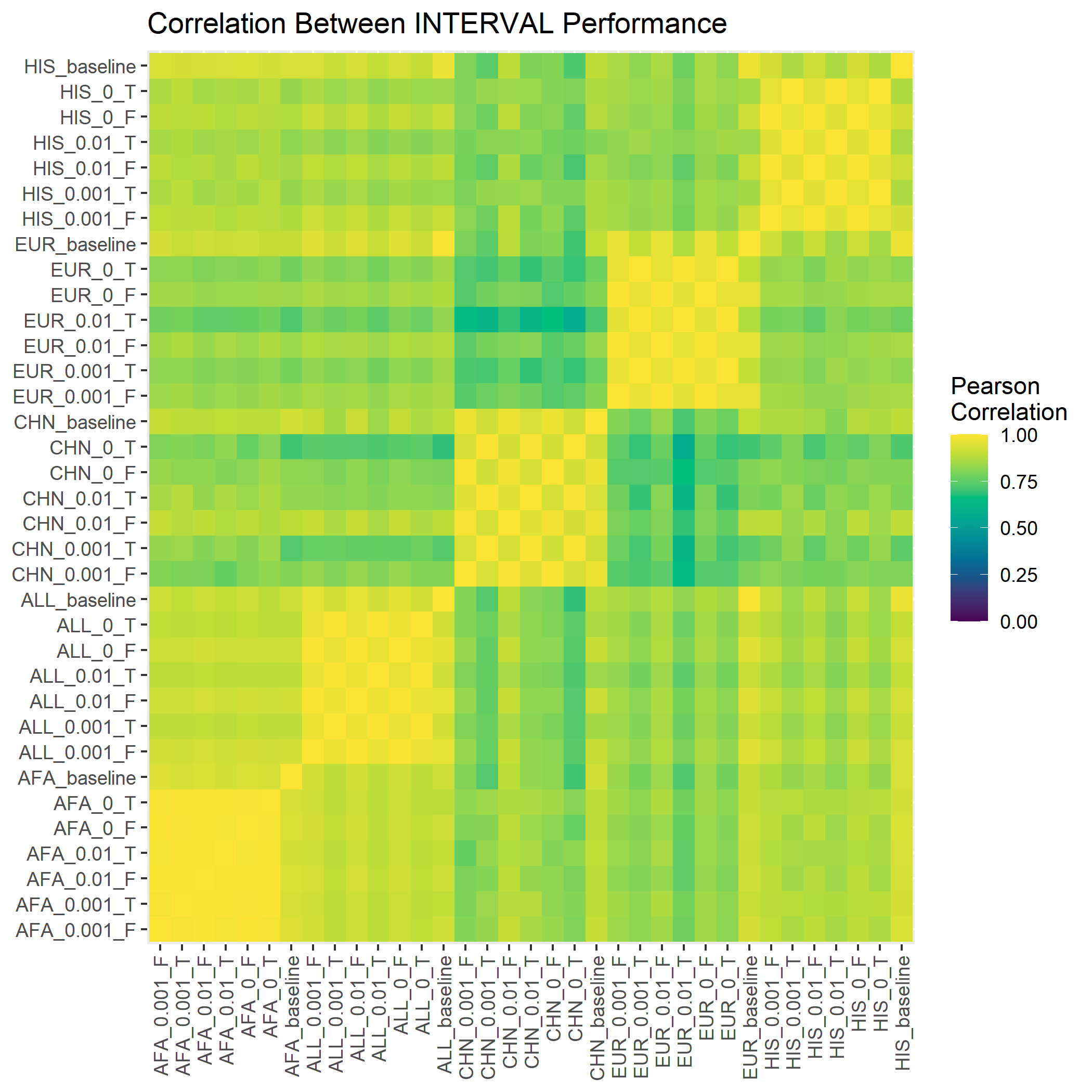

Supplement: S4 Fig — The pairwise Pearson correlations between prediction performance of each model building strategy trained in each TOPMed MESA population. Prediction performance is the Spearman correlation between observed and predicted expression in the independent INTERVAL study. Note, most fine-mapped models within a population had high correlation, with slightly reduced correlations between fine-mapped (LD cluster filtered true) and baseline models. See S1 Table for model notations. (TIF) [file pone.0264341.s004.tif]

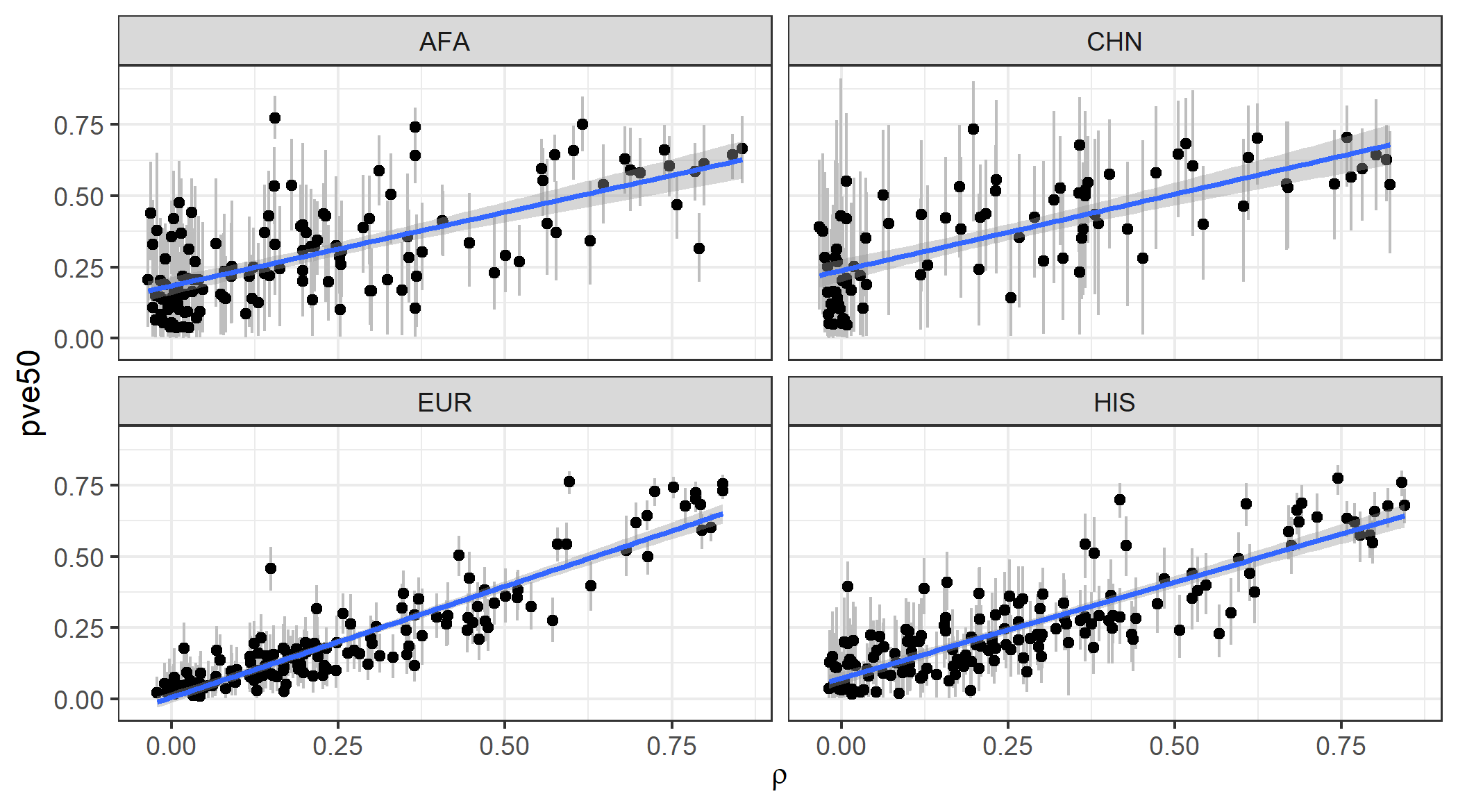

Supplement: S5 Fig — Comparison of the BSLMM PVE (pve50) by cis-SNPs for each protein trait in each population to the prediction performance in INTERVAL (ρ). Gray vertical lines represent the 95% credible set for each PVE estimate and the blue line is the linear regression fit. (TIF) [file pone.0264341.s005.tif]

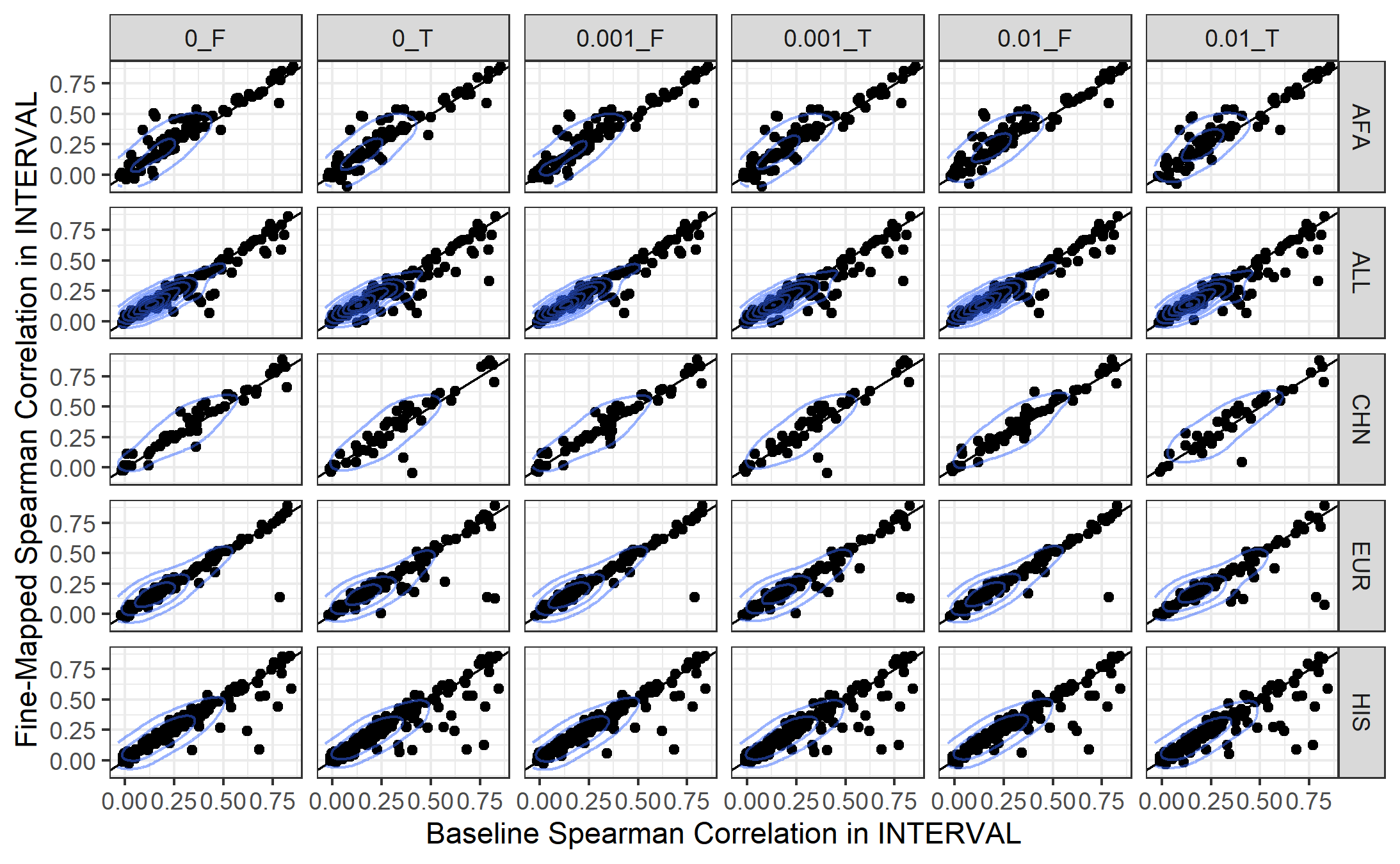

Supplement: S6 Fig — Vertical axis is the fine mapped model performance when predicting in INTERVAL. Horizontal axis is the baseline elastic net model performance when predicting in INTERVAL. Each dot represents a protein that is predicted by both baseline models and fine mapped models. Performance is measured as the Spearman correlation between the measured protein aptamer level and the predicted protein aptamer level. (TIF) [file pone.0264341.s006.tif]

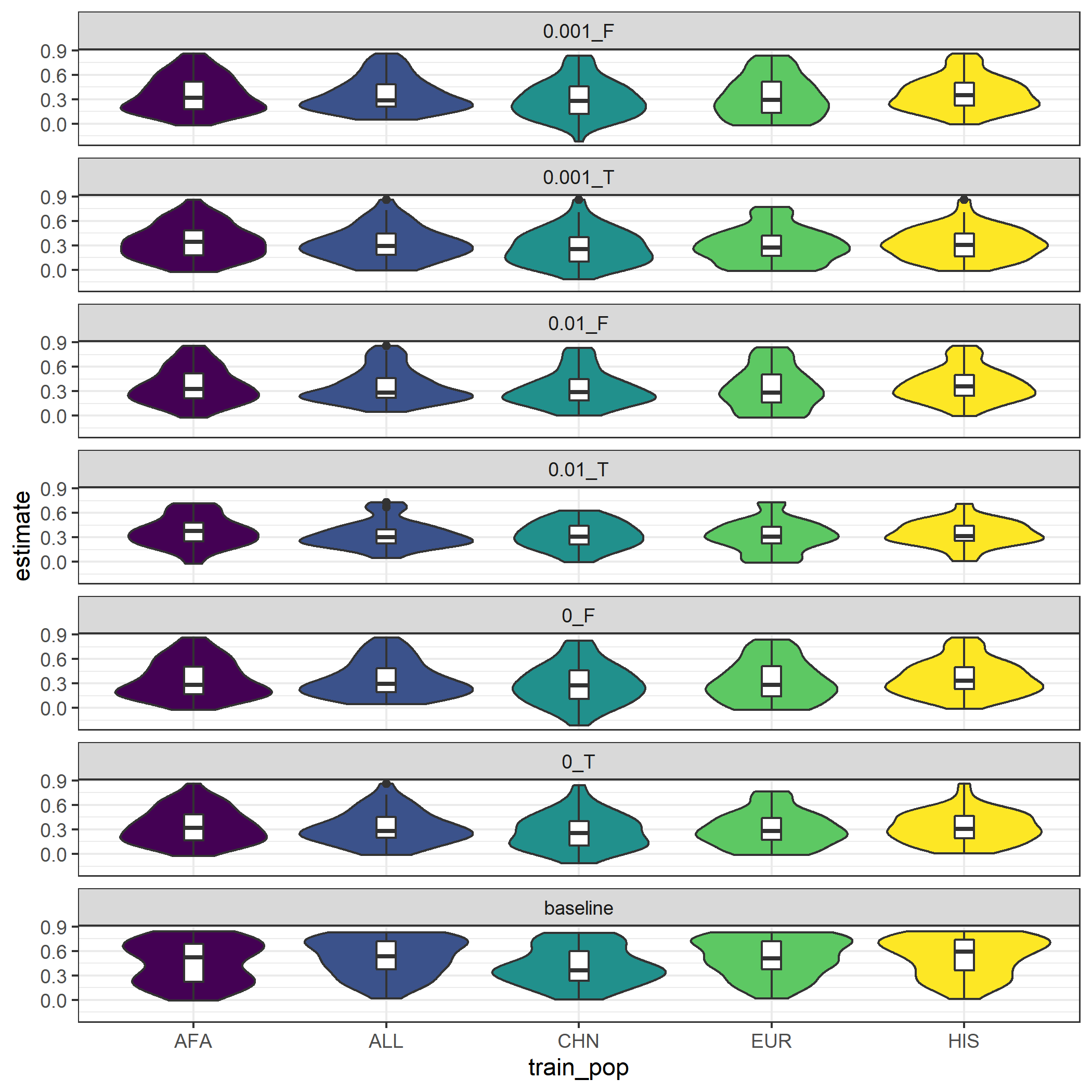

Supplement: S7 Fig — We compare the performance of our different training populations at predicting in INTERVAL, a predominantly European cohort. For a particular model building strategy we first take the intersection of proteins that are predicted by all five training populations and then test for differences in the distribution of Spearman correlations by ANOVA and permuted F-test. We find a significant difference among training populations for our baseline elastic net models (30 proteins, F = 13.30, p = 5.93e-09), 0.001_F models (61 proteins, F = 3.41, p = 0.0098), and 0_F models (59 proteins, F = 3.54, p = 0.0080). (TIF) [file pone.0264341.s007.tif]

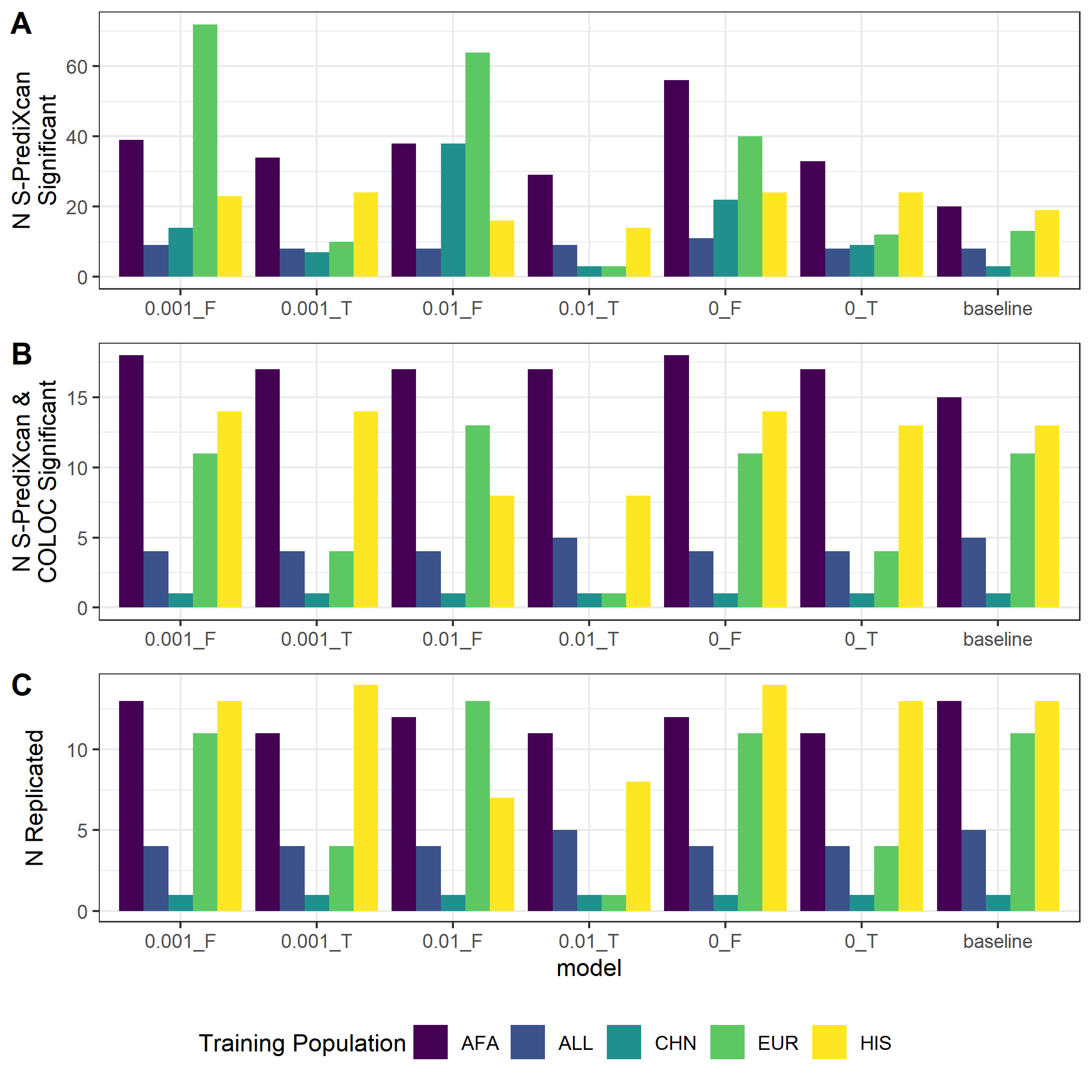

Supplement: S8 Fig — Fine-mapped model sets consistently have a greater number of Bonferroni significant associations than baseline model sets. However when including significant evidence of colocalization by COLOC and replication status as additional significance criteria, baseline has a higher number of significant associations. (TIF) [file pone.0264341.s008.tif]

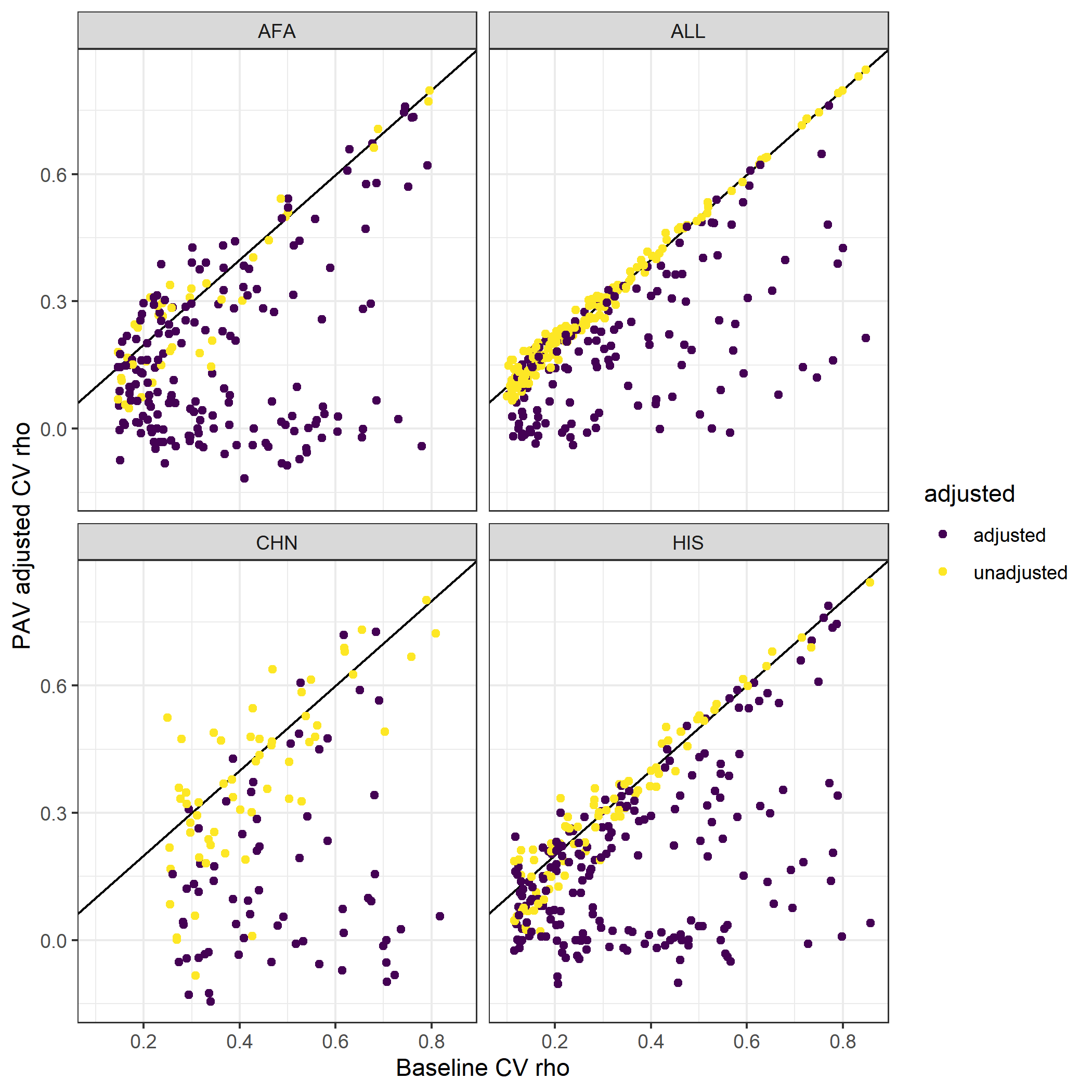

Supplement: S9 Fig — Cross-validated rho within each TOPMed MESA population is plotted on both axes. PAV adjusted model sets are on the Y axis, while standard model sets are plotted on the X axis. Most models were unadjusted for PAVs as the protein does not contain a PAV (yellow points). (TIF) [file pone.0264341.s009.tif]

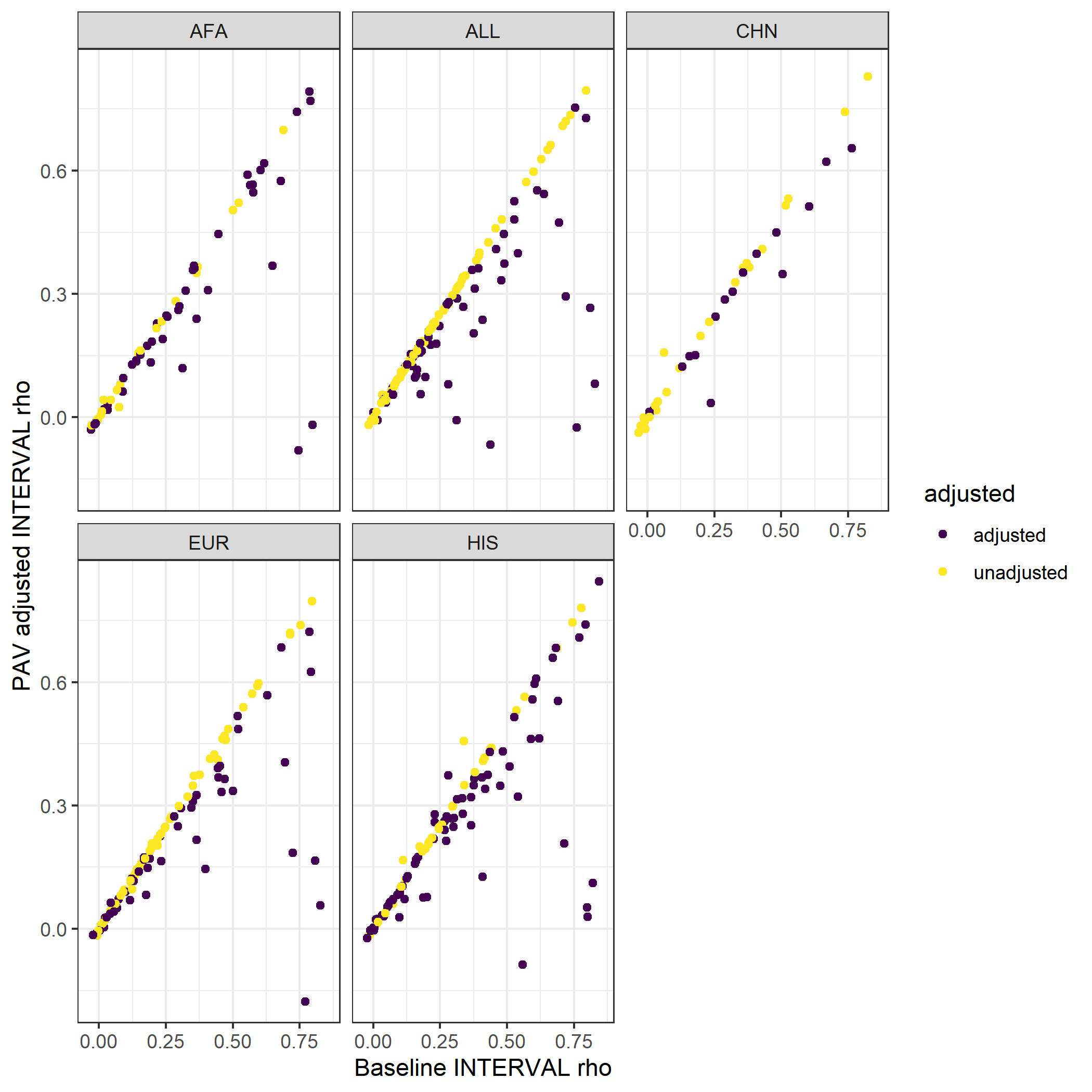

Supplement: S10 Fig — Prediction performance rho in INTERVAL using models built in each TOPMed MESA population is plotted. PAV adjusted model sets are on the Y axis, while standard model sets are plotted on the X axis. Most models were unadjusted for PAVs as the protein does not contain a PAV (yellow points). Most models are either unadjusted (yellow) or have only a small decrease in performance. 7.0% of models had a larger decrease in performance (change in ρ > 0.1), but maintained significance. Not plotted here is the 23.6% of models which are significant in our unadjusted regression, but are no longer significant in our PAV adjusted regression. (TIF) [file pone.0264341.s010.tif]

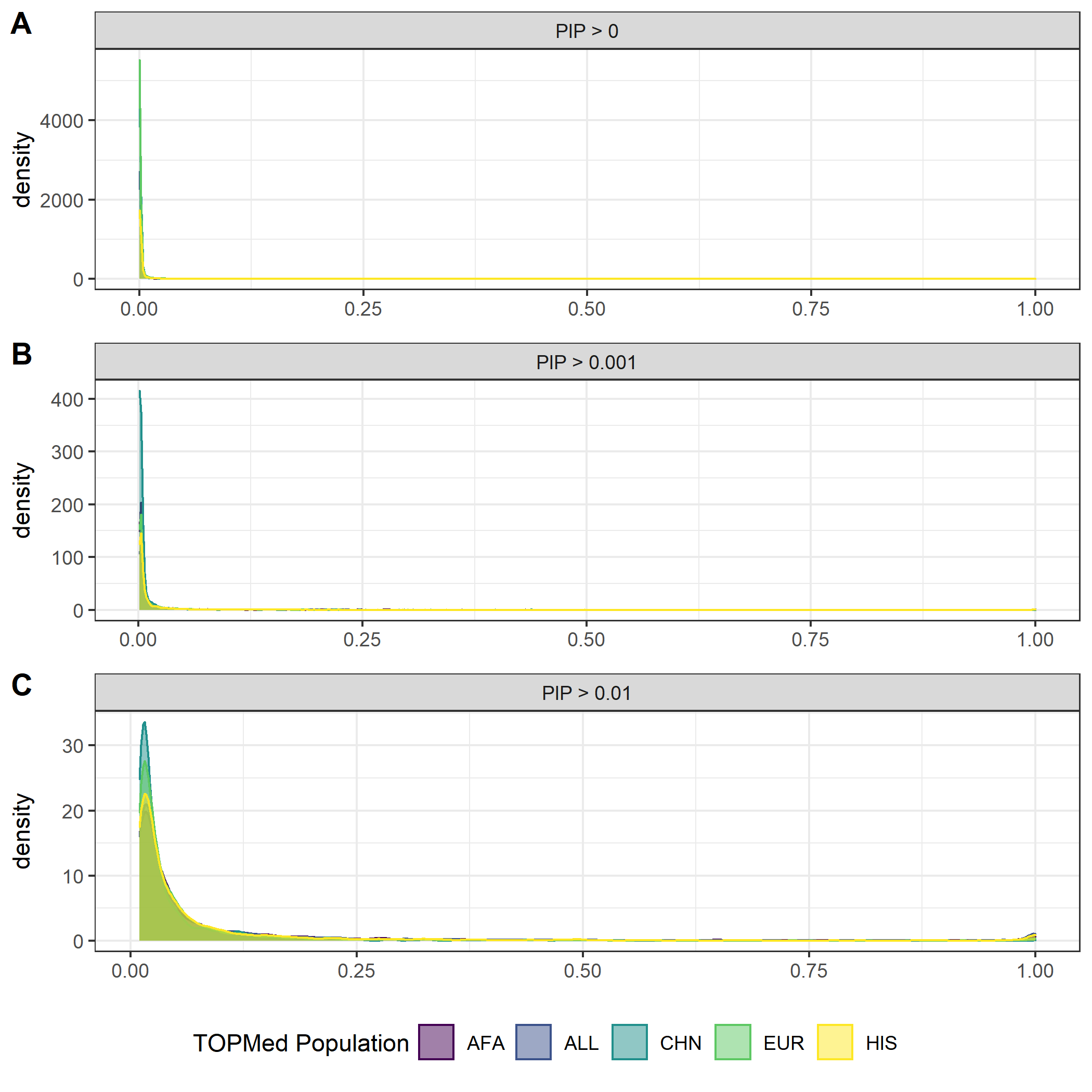

Supplement: S11 Fig — The vast majority of PIPs used to calculate penalty factors in our fine-mapped models are near 0. A) Distribution of PIPs >0 B) PIPs >0.001 C) PIPs >0.01. (TIF) [file pone.0264341.s011.tif]
